# Negative pressure wound therapy for closed incisions in orthopedic trauma surgery: a meta-analysis

**DOI:** 10.1186/s13018-019-1488-z

**Published:** 2019-12-11

**Authors:** Cong Wang, Yiwen Zhang, Hao Qu

**Affiliations:** 1 Department of Orthopaedic Surgery, The Second Affiliated Hospital, Zhejiang University School of Medicine, Hangzhou, 310009 People’s Republic of China; 2 Operating Room, The Second Affiliated Hospital, Zhejiang University School of Medicine, Hangzhou, 310009 People’s Republic of China

**Keywords:** Negative pressure wound therapy, Conventional wound dressings, Surgical site infection, Closed incisions, Orthopaedic trauma, Meta-analysis

## Abstract

**Background:**

This meta-analysis was performed to determine the efficacy of negative pressure wound therapy (NPWT) versus conventional wound dressings for closed incisions in orthopedic trauma surgery.

**Methods:**

A systematic search was performed in PubMed, Embase, and the Cochrane Library databases. The outcome measures included deep surgical site infection (SSI), superficial SSI; wound dehiscence and length of hospital stay. Cochrane collaboration’s tool and the Newcastle–Ottawa Scale (NOS) were used to evaluate literature qualities. Meta-analysis was performed using RevMan 5.3 software.

**Results:**

A total of 6 studies including 2 randomized controlled trials (RCTs) and 4 cohort studies met our inclusion criteria. NPWT resulted in a significantly lower incidence of deep SSI, superficial SSI, and wound dehiscence than conventional wound dressings. However, no statistically significant difference was found in the length of hospital stay.

**Conclusions:**

NPWT appeared to be an efficient alternative to help prevent SSIs and wound dehiscence on closed incisions in orthopedic trauma surgery. Rational use of NWPT should be based on the presence of patient’s condition and risk factors.

## Background

Wound complications within the realm of orthopedic trauma surgery are a major concern. Wound healing is particularly challenging following high-energy trauma, and frequently contribute to postoperative wound dehiscence and surgical site infections (SSIs) [1, 2]. A prospective randomized clinical trial showed an incidence of almost 19% SSIs following high risk lower extremity fracture surgery [3]. SSIs are a serious wound complication leading to increased postoperative morbidity, mortality, length of hospital stay, and economic costs [4]. With the development of new techniques and strategies, attempts are made to manipulate the wound healing process, improve healing rates, and lower the incidence of infectious complications. Examples are antibiotic prophylaxis, multiple-dose administration of prophylaxis, less invasive surgical approaches, and prophylactic negative pressure wound therapy (NPWT).

NPWT as an adjunct to wound healing has been developed more than a decade ago [5]. NPWT has 3 main components that create a negative pressure environment: a vacuum device, a porous dressing, and a connector that allows communication. The porous dressing placed on the wound is a dry, hydrophobic, reticulated polyurethane-ether foam. The wound and porous dressing are sealed via an occlusive adhesive dressing, and communicate with the vacuum device via a connector creating a subatmospheric pressure environment. NPWT promotes wound healing by providing wound coverage, reducing dead space and minimizing tension, increasing blood flow, reducing edema, and constructing an environment that encourages tissue granulation [6–8]. It has been used successfully in open wound management and wound complications following orthopedic surgery. As orthopedists become more familiar with NPWT, they expanded the application in various surgical procedures; for example, it is now being used as postoperative dressing for fasciotomy wounds after compartment release [9]. Recent studies have showed the application of prophylactic NPWT on closed incisions following high-energy lower extremity trauma and total joint arthroplasty [3, 10–12]. These positive results suggest that NPWT may become an adjunct to reduce wound complications for primarily closed incisions in orthopedic trauma surgery, but no clear consensus was achieved based on current researches.

The purpose of this study was to meta-analyze the current evidence in the literature to assess clinical results of NPWT versus conventional wound dressings for closed incisions in orthopedic trauma surgery. The hypothesis was that NPWT would result in less SSIs and wound dehiscence when compared with conventional wound dressings.

## Methods

### Literature search

Two independent reviewers performed this study according to the PRISMA (Preferred Reporting Items for Systematic Reviews and Meta-Analyses) guidelines. The electronic databases of PubMed, Embase, and the Cochrane Library databases were searched from the inception of the database to November 1, 2019. The following search terms was used: (negative pressure wound therapy or negative pressure dressings or vsd or vacuum sealing drainage or vacuum-assisted closure) and (fracture or orthopedic trauma) and incision. The title and abstract were screened for all retrieved citations, and potentially suitable studies received a full-text review. Furthermore, the reference lists from included articles and relevant reviews were assessed to identify additional studies meeting the inclusion criteria.

### Inclusion and exclusion criteria

We identified literature that met the following inclusion criteria: (1) clinical studies comparing NPWT versus conventional wound dressings for closed incisions in orthopedic trauma surgery, including randomized control trials (RCTs), cohort studies, and case-control studies; (2) published in English; (3) outcomes including the incidence of SSIs; and (4) full text of studies available. The exclusion criteria were the following: (1) abstract, letters, editorials, conference articles, case reports, reviews, animal studies, and study protocols, (2) repeated studies and data.

### Data extraction

Two reviewers independently extracted the data from each study using a predefined data sheet, relevant data extracted included first author, year of publication, study design, sample size, age, duration of NPWT treatment, type of fracture, follow-up, and outcome measures. Whenever necessary, we contacted the authors of the studies for the missing data and additional information.

### Assessment of study quality

The methodological quality assessment of the included RCTs was independently assessed by two reviewers using the Cochrane collaboration’s tool [13]. The risk of bias was classified as low, unclear, or high risk. The methodological quality of included cohort studies was assessed according to the Newcastle–Ottawa Scale (NOS) [14]. The NOS uses a star system ranges from zero to nine stars. Studies of high quality were defined as those with scores higher than 6 stars.

### Outcomes measured

The outcomes measured focused on (1) deep SSI; (2) superficial SSI; (3) wound dehiscence; (4) length of hospital stay.

### Statistical analysis

Statistical analysis was performed using RevMan 5.3 for outcome measurements. The estimate of the overall results was showed in forest plot. Odds ratios (OR) with 95% confidence interval (95% CI) were calculated for dichotomous outcomes, and mean difference (MD) with 95% CI were used for continuous outcomes. Heterogeneity among studies was tested using *I*^2^ statistic, and substantial heterogeneity was represented by an *I*^2^ value greater than 50%. If significant heterogeneity was found in the meta-analysis, we used a random effect model; otherwise, we used a fixed effect model. *P* value less than 0.05 was considered to be statistically significant.

## Results

### Literature search

**Fig. 1:**
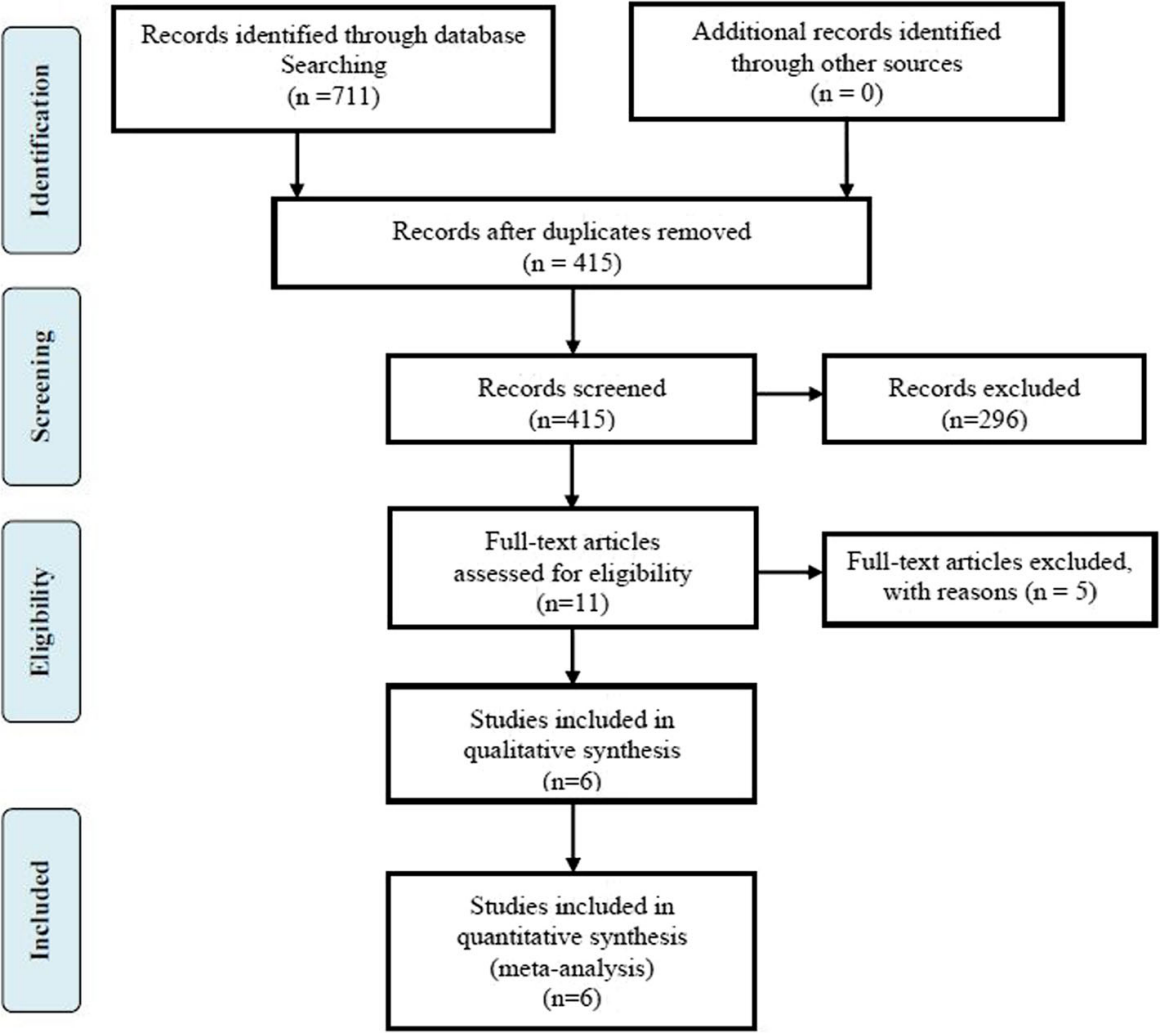
The flow chart of literature screening

The initial electronic databases search yielded 711 relevant articles. Manual searching of relevant references did not add additional studies. After both duplicate checking and title and abstract reviewing, 700 studies were excluded. The remaining 11 studies were subjected to full-text screen. Ultimately, two RCTs and four cohort trials were eligible for meta-analysis [3, 10, 15–18]. A flowchart of the literature search is shown in Fig. 1.

### Characteristics and quality assessment of the eligible studies

The characteristics of the six included studies are listed in Table 1. The studies were published between 2010 and 2018. The two RCTs and four cohort trials involved a total of 859 patients. A total of 500 patients used NPWT after the surgeries, whereas 359 applied conventional wound dressings. A great majority of the included patients suffered from low extremity and acetabular fractures. The risk of bias assessment of two RCTs are illustrated in Table 2. The methodological quality of four cohort trials assessed with the NOS are shown in Table 3.

**Table 1:** Characteristics of the studies included in the meta-analysis

Study(year)	Study Design	Sample size		Age, mean ± SD, y		Duration of NPWT treatment	Type of fracture	Follow-up	Outcome measures
		NPWT	Conventional dressings	NPWT	Conventional dressings				
Cooper (2018) [15]	RCS	27	40	76.3(58–91)	73.2(43–91)	7 days	Periprosthetic fracture	90 days	Incisional complications, deep SSI, and reoperations
Crist (2017) [16]	RCT	33	33	44.2(19–87)	43.2(18–92)	>2 days	Acetabular fracture	Nr	Deep infection
Dingemans (2018) [17]	PCS	53	47	43.9 ± 15.6	42.2 ± 14.6	7 days	Ankle, talus, calcaneus, mid-foot fracture	2-4 weeks	Surgical site infection, wound dehiscence
Reddix (2010) [10]	RCS	235	66	40.2 (11–75)	40.4 (16–80)	1-3 days	Acetabular fracture	16.2months/37.3months	Deep wound infection, wound dehiscence
Stannard (2012) [3]	RCT	130	119	43 (18–80)	43(18-80)	59h(21-213)	Tibial plateau, pilon, and calcaneus fractures	Nr	Acute infections, late infections, and wound dehiscence
Zhou (2016) [18]	RCS	22	54	59.1 ± 4.3	57.2 ± 6.2	7 days	Ankle fracture	Nr	Superficial SSI, deep SSI, length of hospital stay and hospital costs

*RCS* retrospective cohort study, *PCS* prospective cohort study, *Nr* not reported

**Table 2:** Risk of bias assessment of randomized controlled trials

Study	Randomization	Allocation concealment	Blinding of participants	Blinding of outcome assessment	Incomplete outcome data	Selective outcome reporting	Other bias
Crist (2017) [16]	Low	Unclear	Unclear	Unclear	Low	Low	Unclear
Stannard (2012) [3]	Low	Unclear	Unclear	Unclear	Low	Low	Unclear

**Table 3:** Quality assessment according to the Newcastle–Ottawa scale

Study	Selection	Comparability	Exposure	Total score
Cooper (2018) [15]	3	2	3	8
Dingemans (2018) [17]	3	2	2	7
Reddix (2010) [10]	3	1	3	7
Zhou (2016) [18]	3	2	2	7

### Deep SSI

**Fig. 2:**
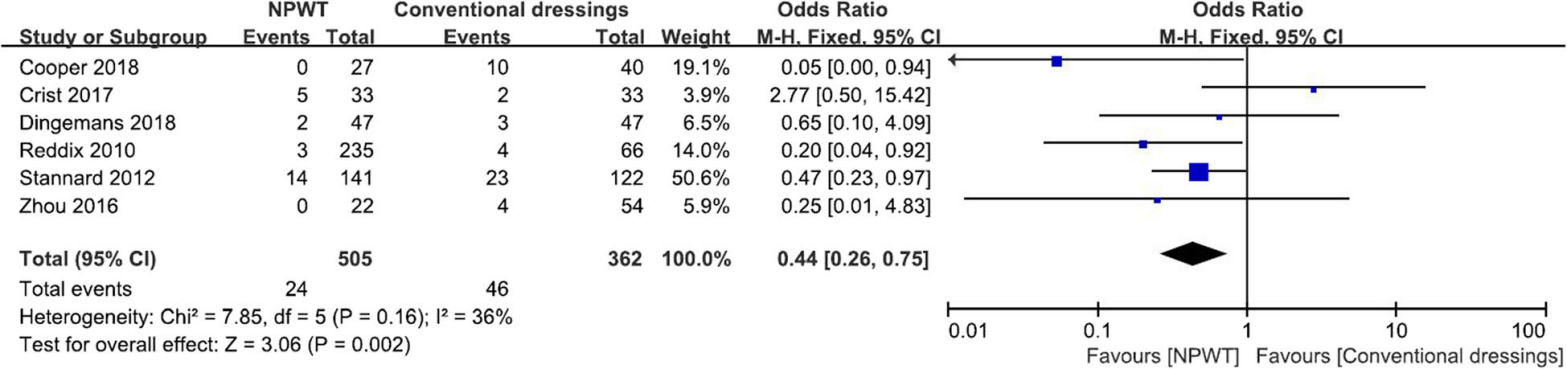
Forest plot showing deep SSI

Deep SSIs were reported in 6 studies [3, 10, 15–18], with 505 NPWT and 362 conventional wound dressings. The NPWT resulted in 4.8% of patients having deep SSIs, compared with conventional wound dressings where 12.7% of patients had deep SSIs. There was a statistically significant difference in favor of the NPWT (OR, 0.44; 95% CI, 0.26–0.75; *I*^2^ = 36%; *P* = 0.002; Fig. 2).

### Superficial SSI

**Fig. 3:**
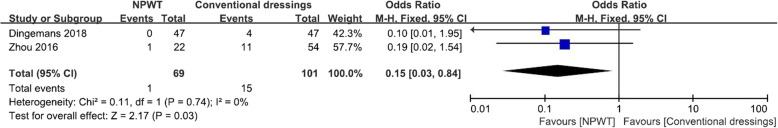
Forest plot showing superficial SSI

Superficial SSIs were reported in 2 studies [17, 18], with 69 NPWT and 101 conventional wound dressings. The NPWT resulted in 1.4% of patients having superficial SSIs, compared with conventional wound dressings where 14.9% of patients had superficial SSIs. There was a statistically significant difference in favor of the NPWT (OR, 0.15; 95% CI, 0.03–0.84; *I*^2^ = 0%; *P* = 0.03; Fig. 3).

### Wound dehiscence

**Fig. 4:**
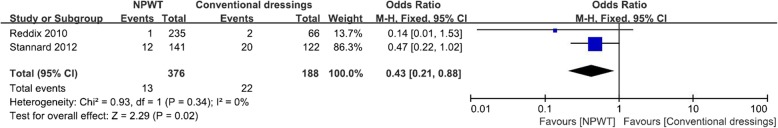
Forest plot showing wound dehiscence

Wound dehiscence was reported in 2 studies [3, 10], with 376 NPWT and 188 conventional wound dressings. The NPWT resulted in 3.5% of patients having wound dehiscence, compared with conventional wound dressings where 11.7% of patients had wound dehiscence. There was a statistically significant difference in favor of the NPWT (OR, 0.43; 95% CI, 0.21–0.88; *I*^2^ = 0%; *P* = 0.02; Fig. 4).

### Length of hospital stay

**Fig. 5:**
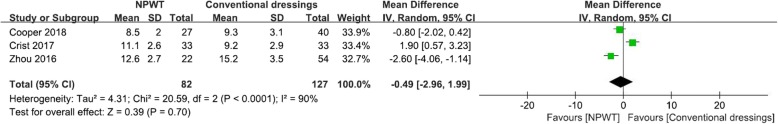
Forest plot showing length of hospital stay

Length of hospital stay was reported in 3 studies [15, 16, 18], comprising 82 NPWT and 127 conventional wound dressings. The NPWT resulted in a mean length of hospital stay of 10.65 days, compared with the conventional wound dressings where mean length of hospital stay was 11.78 days. There was no statistically significant difference between the techniques (MD, − 0.49; 95% CI, − 2.96–1.99; *I*^2^ = 90%; *P* = 0.7; Fig. 5).

## Discussion

The successful use of NPWT on open wound management led some orthopedists to expand the application of NPWT for some closed incisions. A recent consensus panel recommended the use of NPWT on patients who are at high risk of postoperative wound complications [19]; however, these recommendations have been challenged by the results of more recent studies in orthopedic trauma [16, 17]. Therefore, we performed this meta-analysis to compare NPWT and conventional wound dressings for closed incisions in orthopedic trauma surgery. The most important finding of present meta-analysis was that NPWT significantly reduced the incidence of deep SSI, superficial SSI, and wound dehiscence, and no increased length of hospital stay was identified.

Regarding the wound complications, previous systematic review and meta-analysis showed that NPWT can reduce the risk of infection of the patients in treatment of open fractures and accelerate the wound healing process [20]. In open wounds, NPWT promotes wound healing by facilitating removal of excess interstitial fluid, reducing edema, enhancing tissue growth, and expansion [6, 8]. In closed incisions, NPWT functions to promote drainage, improve lymphatic flow, decrease hematoma, and seroma formation, and it reduces relative motion at the surgical site and decreases lateral tension across the incision line [21–23]. Recent clinical studies suggest that NPWT can be a prophylactic treatment to decrease the incidence of infection in high-risk patients following lower extremity fractures as well as following total joint arthroplasty [24, 25]. Consistently, the present meta-analysis indicated that NPWT significantly reduced the incidence of deep surgical site infection, superficial surgical site infection, and wound dehiscence on closed incisions in orthopedic trauma surgery. However, there was no statistically significant difference in the length of hospital stay. For wound, complication is not the only determinant. The duration of NPWT treatment may also influence the hospital stay. And the sample size may be too small to reflect significant difference between the groups.

An obvious and important advantage of NPWT is that it needs less dressing changes compared with conventional wound dressings. NPWT reduces the strain on doctor and nursing staff, and it is particularly obvious in obese patients or in special wound locations, such as the popliteal fossa, buttocks, or groin. To some extent, use of NPWT is beneficial in the prevention of wound infection as each dressing change is a potential opportunity of contamination of the wound. Therefore, NPWT is suitable for the patients who are sent to the intensive care unit during the immediate postoperative period. Furthermore, patients were satisfied with the NPWT as it provides a cleaner wound environment, and they did not have to take care of the surgical incision.

In current modern healthcare environment, it is also important to consider the economic factors when we make treatment decisions. The costs of NPWT have been estimated to be less than $500 per patient [3], but the health care costs associated with postoperative SSIs can be enormous [26, 27]. Therefore, in patients at high risk for wound complications, it would be reasonable and cost-effective to use NPWT for closed incisions in orthopedic trauma surgery. Furthermore, the use of NPWT did not increased the length of hospital stay.

Although the current application of NPWT for closed incisions in orthopedic trauma surgery has yielded some satisfactory results, it does not mean that NPWT should be used for all orthopedic trauma surgeries. Rational use of NWPT should be based on the presence of patient’s condition and risk factors [19]. The fractures involved in present study are calcaneus, pilon, ankle, tibial plateau, and acetabular fractures, which are frequently accompanied with high likelihood of prolonged wound drainage and postoperative wound swelling [11]. Those patients are at a high risk of SSIs and soft tissue healing problems after the surgeries. And this problem is further aggravated if the patient has the most common patient-related risk factors, including obesity, diabetes mellitus, tobacco use, and prolonged surgical time [28–31]. Therefore, it is reasonable that current study obtained the positive results about the use of NPWT for closed incisions. It is important to note that study to support its widespread application in all cases in orthopedic trauma surgery is lacking, large, and well-designed RCTs are needed in the future to validate the efficacy and safety of NPWT.

Nonetheless, some limitations in the present meta-analysis should be noted. (1) Only four cohort studies and two RCTs were included in the present meta-analysis, and the sample size was relatively small, which might lower the evidence level. (2) The duration of NPWT treatment of the included studies were inconsistent. (3) The fractures involved in present study are lower extremity and acetabular fractures, which are at a high risk of infection, and soft tissue healing problems after surgeries, so the outcome should be treated cautiously.

## Conclusion

In conclusion, NPWT appeared to be an efficient alternative to help prevent SSIs and wound dehiscence on closed incisions in orthopedic trauma surgery. Rational use of NWPT should be based on the presence of patient's condition and risk factors. More large multi-center and high-quality RCTs are required for further research [32].

## Data Availability

All data generated or analyzed during this study are included in published articles.

## Abbreviations

CI: Confidence interval
MD: Mean difference
NOS: Newcastle–Ottawa Scale
NPWT: Negative pressure wound therapy
OR: Odds ratios
RCTs: Randomized controlled trials
SSI: Surgical site infection
